# Serum microRNA profiles in children with autism

**DOI:** 10.1186/2040-2392-5-40

**Published:** 2014-07-30

**Authors:** Mahesh Mundalil Vasu, Ayyappan Anitha, Ismail Thanseem, Katsuaki Suzuki, Kohei Yamada, Taro Takahashi, Tomoyasu Wakuda, Keiko Iwata, Masatsugu Tsujii, Toshirou Sugiyama, Norio Mori

**Affiliations:** 1 Department of Psychiatry, Hamamatsu University School of Medicine, 1-20-1 Handayama, Higashi-ku, Hamamatsu 431-3192, Japan; 2 Research Center for Child Mental Development, Hamamatsu University School of Medicine, Hamamatsu, 1-20-1 Handayama, Higashi-ku, Hamamatsu 431-3192, Japan; 3 Research Center for Child Mental Development, University of Fukui, 23-3 Matsuokashimoaizuki, Eiheiji, Fukui 910-1193, Japan; 4 Faculty of Sociology, Chukyo University, 101 Tokodachi, Kaizu-cho, Toyota 470-0393, Japan; 5 Department of Child and Adolescent Psychiatry, Hamamatsu University School of Medicine, 1-20-1 Handayama, Higashi-ku, Hamamatsu 431-3192, Japan

**Keywords:** Autism spectrum disorder, microRNA, complementary DNA, microarray, quantitative PCR

## Abstract

**Background:**

As regulators of gene expression, microRNAs (miRNAs) play a key role in the transcriptional networks of the developing human brain. Circulating miRNAs in the serum and plasma are remarkably stable and are suggested to have promise as noninvasive biomarkers for neurological and neurodevelopmental disorders. We examined the serum expression profiles of neurologically relevant miRNAs in autism spectrum disorder (ASD), a complex neurodevelopmental disorder characterized by multiple deficits in communication, social interaction and behavior.

**Methods:**

Total RNA, including miRNA, was extracted from the serum samples of 55 individuals with ASD and 55 age- and sex-matched control subjects, and the mature miRNAs were selectively converted into cDNA. Initially, the expression of 125 mature miRNAs was compared between pooled control and ASD samples. The differential expression of 14 miRNAs was further validated by SYBR Green quantitative PCR of individual samples. Receiver-operating characteristic (ROC) analysis was used to evaluate the sensitivity and specificity of miRNAs. The target genes and pathways of miRNAs were predicted using DIANA mirPath software.

**Results:**

Thirteen miRNAs were differentially expressed in ASD individuals compared to the controls. MiR-151a-3p, miR-181b-5p, miR-320a, miR-328, miR-433, miR-489, miR-572, and miR-663a were downregulated, while miR-101-3p, miR-106b-5p, miR-130a-3p, miR-195-5p, and miR-19b-3p were upregulated. Five miRNAs showed good predictive power for distinguishing individuals with ASD. The target genes of these miRNAs were enriched in several crucial neurological pathways.

**Conclusions:**

This is the first study of serum miRNAs in ASD individuals. The results suggest that a set of serum miRNAs might serve as a possible noninvasive biomarker for ASD.

## Background

Autism spectrum disorder (ASD) refers to a group of heterogeneous neurodevelopmental disorders characterized by impairments in communication and social interaction, and restricted, repetitive and stereotypic patterns of behavior [1]. According to a recent estimate, 1 in 88 individuals has ASD [2]. ASD is largely genetic in origin, with most data supporting a polygenic epistatic model [3,4]. However, owing to the heterogeneous nature of this disorder, classical genetic studies have not necessarily been successful in identifying suitable candidate genes for ASD. In addition to the genetic factors, environmental factors also play a vital role in predisposing individuals to ASD [5]. In recent years, epigenetic mechanisms, which act at the interface of genes and the environment, have been identified as a potential contributor to the pathogenesis of several neurodevelopmental abnormalities such as ASD [6]. Epigenetic factors control heritable changes in gene expression without changing the DNA sequence [7].

MicroRNAs (miRNAs) have recently emerged as prominent epigenetic regulators of a variety of cellular processes, including differentiation, apoptosis and metabolism [8]. miRNAs are a class of small (approximately 21 nucleotides) noncoding transcripts that can modulate cellular messenger RNA (mRNA) and protein levels by interacting with specific mRNAs, usually at the 3′ untranslated region (UTR), resulting in mRNA degradation or repression of translation [9,10], through partial sequence complementation [11]. MiRNAs are abundantly present in the brain, and have been found to play crucial roles in several facets of brain function, particularly in neuronal plasticity and neuronal development [12].

So far, three studies have determined the expression of miRNAs in lymphoblastoid cell cultures of ASD patients, and one study has identified dysregulated miRNAs in the cerebellar cortex of ASD patients. Abu-Elneel *et al*. first observed an altered expression of miRNAs and their brain-specific targets in the postmortem cerebellar cortices of autism subjects [13]. Studies in lymphoblastoid cells have implicated brain-related miRNAs and their targets in the pathophysiological conditions underlying autism [14-16].

MiRNAs have also been found to be present in the extracellular fluids such as plasma, serum, saliva, and urine of humans in detectable concentrations [17-20]. In particular, serum miRNAs, which may be derived from circulating blood cells, are known to be remarkably stable, reproducible and resistant to the actions of RNase [21], suggesting the potential efficacy of serum miRNAs as noninvasive biomarkers for ASD. Therefore, in the present study we performed miRNA expression profiling of serum samples from individuals with ASD who had never received drug treatment.

## Methods

This study was approved by the Ethics Committee of Hamamatsu University School of Medicine, Japan. A detailed description of the study was given to all participants and their parents before enrollment. Blood samples were collected from the donors after obtaining written informed consent.

### Subjects

In this study, we included 55 subjects with ASD (age = 11.29 ± 2.45 years (mean ± SD); range = 6 to 16 years; 48 males and 7 females) and 55 age- and sex-matched typically developed control subjects (age = 11.3 ± 2.37; range = 6 to 16; 41 males and 14 females). There were no significance differences in the age (*P* = 0.9685) or sex (*P* = 0.089) distribution between the control and ASD groups (Table 1). All of the participants were Japanese. The diagnosis of ASD was made on the basis of the Diagnostic and Statistical Manual, Fourth Edition, Text Revision (DSM-IV-TR; American Psychiatric Association, 2000) criteria. The Autism Diagnostic Interview-Revised (ADI-R) [22] was conducted by experienced child psychiatrists who are licensed to use the Japanese version of the ADI-R. Participants having comorbid psychiatric illnesses were excluded by means of the Structured Clinical Interview for DSM-IV (SCID) [23]; any additional psychiatric or neurological diagnosis was also excluded. None of the participants had received any drug treatment for ASD.

**Table 1:** Clinical and/demographic variables of individuals with autism and of control subjects

**Clinical/demographic**	**Autism (n = 55)**	**Control (n = 55)**	***P *** **value**
Age	11.29 ± 2.45 (6 to 16)	11.31 ± 2.37 (6 to 16)	0.9685^a^
Sex			
Male	48	41	0.089^b^
Female	7	14	
ADI-R			
Domain A score, social	17.7 ± 7.62 (10 to 29)		
Domain BV score, communication	12.8 ± 5.62 (8 to 25)		
Domain C score, stereotype	4.1 ± 2.84 (3 to 12)		

Values are expressed as the mean ± SD (range).^a^*t*-test, ^b^chi-square test. *ADI-R*, Autism Diagnostic Interview-Revised.

Typically developed individuals (control group) were recruited through advertisements in local newspapers. Control group participants underwent a comprehensive assessment of their medical history; those with neurological or other medical disorders were excluded. The SCID was also conducted to screen all participants for any past or present mental illness. None of the control participants were diagnosed with any neuropsychiatric condition.

### Serum separation

Blood samples were collected between 11:00 am and noon from each subject by venipuncture, and the samples were kept for 30 min at room temperature. All protocols for serum separation were completed within 1 h of drawing blood. Serum was separated by centrifugation at 3,500 rpm for 10 min at room temperature. Hemolyzed samples were excluded from the study at this stage. The clear supernatant was collected into RNase/DNase-free microfuge tubes in 200 μl aliquots and stored at -80°C until use.

### RNA extraction and cDNA synthesis

Total RNA, including miRNA, was extracted from 200 μl serum by using an MiRNeasy Serum/Plasma Kit (QIAGEN GmbH, Hilden, Germany) in accordance with the manufacturer’s protocol. Briefly, five volumes of QIAzol lysis reagent was added to the sample; a synthetic spike-in control, *Caenorhabditis elegans* miR-39 (1.6 × 10^8^ copies/μl), was added to the lysed samples for internal normalization. After adding an equal volume of chloroform, the samples were centrifuged for 15 min at 12,000 g at 4°C. The upper aqueous phase was mixed with 1.5 volumes of 100% ethanol, transferred to a spin column, centrifuged, washed, and eluted in 14 μl RNase-free water.

Two microliters of each RNA sample was used for cDNA synthesis using the miScript ІІ RT kit (QIAGEN). The reverse-transcription reaction mix (20 μl) was prepared using Hispec buffer (for selective conversion of mature miRNAs into cDNA), nucleics mix, RT mix and RNase-free water. The reaction mixture was incubated for 60 min at 37°C, followed by denaturation for 5 min at 95°C. Each cDNA was further diluted to 220 μl with RNase-free water and stored at -20°C until use.

### microRNA screening

Initial screening was done using the Human Neurological Development & Disease miRNA PCR array (SABiosciences, Frederick, MD, USA), which contains 84 miRNA assays, and a custom-made array (SABiosciences) with 41 miRNA assays (see Additional files 1 and 2). For this purpose, 40 samples were chosen from 55 ASD subjects at random, and 40 matched control samples were selected. All the miRNAs included in the arrays have previously been reported to play a role in various aspects of brain development and function and/or in several neuropsychiatric conditions such as ASD. Both arrays included *C. elegans* miR-39 primer assays for internal normalization, snoRNA/snRNA (SNORD48, SNORD61, SNORD68, SNORD72, SNORD95, SNORD96A and RNU6-2) PCR control assays, positive PCR control (PPC) assays, and miRNA reverse transcription control (miRTC) assays.

RNA from age- and sex-matched control (n = 40) and ASD (n = 40) samples were pooled separately to generate four pools per group, with each pool consisting of 10 samples. Then, cDNA prepared from 3 μl of the pooled RNA was used for array screening. The expression of miRNAs was detected and quantified by means of SYBR Green reverse-transcription quantitative PCR performed with an ABI PRISM 7900 Sequence Detection System (Applied Biosystems, Foster City, CA, USA).

#### Normalization

The data sets were first calibrated using a *C. elegans* miR-39 assay, which detected the spike-in control that was added to the serum samples during RNA extraction. This calibration would have resolved any differences in recovery that may have occurred during the purification procedure, or any differences in amplification efficiency. Due to the very low expression of snoRNA/snRNA PCR controls in the serum, three alternate normalization strategies, as recommended by the manufacturer, were used for data normalization. These were (i) normalization to the whole plate Ct mean, (ii) normalization to the plate Ct mean of commonly expressed (Ct < 30) miRNAs, and (iii) normalization to the Ct mean of at least four invariant miRNAs with little (<1) Ct variation between samples. In the third strategy, miR-125b-5p, miR-126-5p, miR-140-5p and miR-191-5p were chosen for the data normalization of the neurological array, whereas miR-103a-3p, miR-21-5p, miR-23a-3p, and miR-25-3p were chosen for the custom array. Consistent results were obtained using all three strategies.

#### Data analysis

An Excel-based miRNA PCR Array Data Analysis tool (SABiosciences; http://pcrdataanalysis.sabiosciences.com/mirna) was used for data analysis. SABiosciences makes use of the ΔΔCt method for the relative quantification of miRNAs. Student’s *t*-test was used to examine any differential expression of miRNAs between the ASD and control groups; values of *P* <0.05 were considered to indicate statistical significance (GEO Accession Number: GSE58850).

### Quantitative reverse-transcription PCR

SYBR Green qPCR, performed on an ABI PRISM 7900 Sequence Detection System (Applied Biosystems), was used for the validation of differentially expressed miRNAs (the accession ID and mature miRNA sequence are given in Additional file 3). In this validation experiment, all the samples (55 subjects with autism and 55 controls) were examined individually. Ten microliters of qPCR reaction mixture was prepared with a universal primer, primer assay and RNase-free water. All the qPCR reactions were performed in triplicate with the following cycling conditions: 95°C → 15 min, followed by 40 cycles of 94°C → 15 sec, 55°C → 30 sec and 70°C → 30 sec.

The Ct values of nine miRNAs (miR-101-3p, miR-106b-5p, miR-151a-3p, miR-195-5p, miR-19b-3p, miR-27a-3p, miR-320a, miR-328, and miR-489) were in the range of 25–30, while the remaining five miRNAs (miR-130a-3p, miR-181b-5p, miR-433, miR-572, and miR-663a) had Ct values in the range of 30 to 35.

#### Normalization

A randomly chosen control sample was amplified in each plate and used as an interplate calibrator to correct for the experimental differences among consecutive PCR runs. The qPCR data was first subjected to interplate calibration, followed by *C. elegans* miR-39 (detection of the spike-in control) calibration. The expression of miR-16, an miRNA highly abundant in the red blood cells, was analyzed in each sample to examine the extent of hemolysis in the serum. Any sample that showed significant hemolysis (the value of Ct invariant miRNA - Ct miR16 was greater than 5) was omitted from further analyses. Finally, the qPCR data were normalized to the average of three invariantly expressed miRNAs, let-7a, miR-191-5p and miR-103a-3p. Among these, miR-191-5p and miR-103a-3p were selected on the basis of their performance as invariant miRNAs in the neurological array and custom array, respectively, while let-7a has been widely reported as an invariant miRNA in blood. The fold change in gene expression between the control and ASD groups was determined by the ΔΔCt method of relative quantification.

#### Statistical analysis

All statistical calculations were performed with PASW Statistics 18 software (IBM, Tokyo, Japan). Student’s *t*-test and chi-square test were used to examine any variability in the distribution of age and sex, respectively, across the control and ASD groups. Any differential expression of miRNAs between the control and ASD groups was determined by Mann–Whitney test. The relationship between the expression of miRNA and ADI-R subscores was evaluated by Spearman’s correlation coefficient. Analysis of covariance (ANCOVA) was used to control for potential covariates such as age and sex. Receiver Operating Characteristics (ROC) curve analysis was used for evaluating the diagnostic power of miRNAs.

### Enrichment pathway analysis and target gene prediction

The DIANA mirPath v2.0 (http://diana.cslab.ece.ntua.gr/pathways/) functional annotation tool was used to predict the target genes and altered pathways of differentially expressed miRNAs. This tool predicts the miRNA targets based on DIANA-microT-CDS and/or experimentally verified targets from TarBase v6 (manually curated, experimentally validated miRNA-gene interactions database).

## Results

### microRNA screening

**Figure 1:**
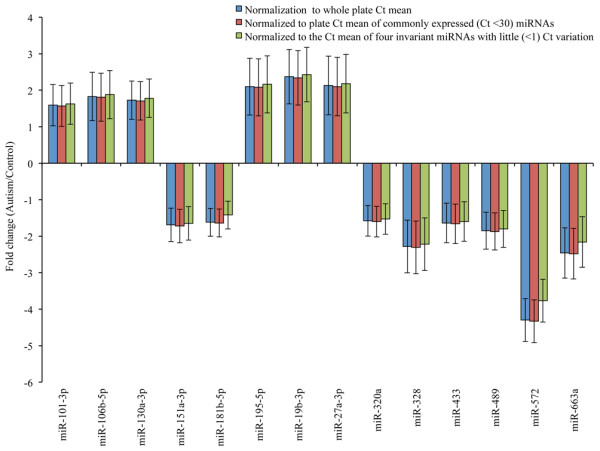
**Results from preliminary screening experiments.** The Ct values obtained were normalized and calculated by three different strategies. miR-151a-3p, miR-181b-5p, miR-320a, miR-328, miR-433, miR-489, miR-572 and miR-663a were downregulated while miR-101-3p, miR-106b-5p, miR-19b-3p, miR-195-5p, miR-130a-3p and miR-27a-3p were upregulated. Bars represent the fold change in subjects with autism as compared to controls.

Ct values of the PPC controls were 19 ± 2 across all samples, indicating the uniformity of reaction conditions. The differences between the Ct values of PPC and miRTC were calculated as <7, indicating that there was no inhibition of the reverse-transcription reaction.In the preliminary array screening, we observed an altered expression of 14 miRNAs in the ASD samples compared to those of controls (Figure 1). MiR-151a-3p, miR-181b-5p, miR-320a, miR-328, miR-433, miR-489, miR-572 and miR-663a were downregulated, while miR-101-3p, miR-106b-5p, miR-19b-3p, miR-195-5p, miR-130a-3p and miR-27a-3p were upregulated.

### Confirmation with quantitative PCR

**Figure 2:**
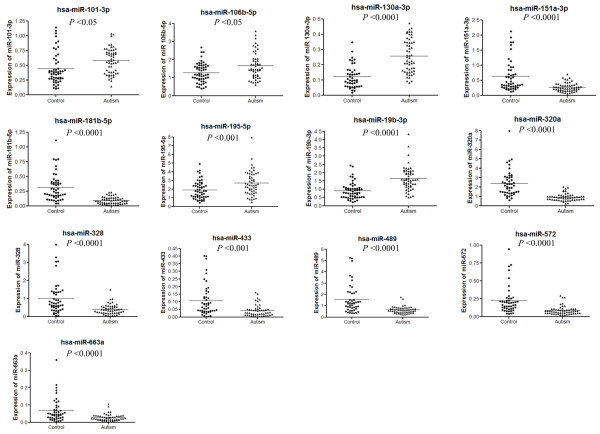
Scatter plot showing the differential expression of 13 microRNAs in autistic subjects.

The differential expression of the 14 miRNAs was further validated by SYBR Green qPCR. We observed consistent results for all miRNAs except miR-27a-3p (Figure 2). miR-151a-3p (ΔΔCt = -2.01, *P* = 8.29E-06), MiR-181b-5p (ΔΔCt = -3.39, *P* = 1.04E-10), miR-320a (ΔΔCt = -2.47, *P* = 5.02E-12), miR-328 (ΔΔCt = -2.28, *P* = 4.33E-06), miR-433 (ΔΔCt = -2.33, *P* = 0.0001), miR-489 (ΔΔCt = -2.10, *P* = 1.25E-06), miR-572 (ΔΔCt = -2.47, *P* = 2.66E-08) and miR-663a (ΔΔCt = -2.06, *P* = 0.00002) were downregulated, while miR-101-3p (ΔΔCt = 1.43, *P* = 0.003), miR-106b-5p (ΔΔCt = 1.30, *P* = 0.008), miR-130a-3p (ΔΔCt = 2.35, *P* = 1.89E-09), miR-195-5p (ΔΔCt = 1.43, *P* = 0.0016) and miR-19b-3p (ΔΔCt = 1.87, *P* = 6.88E-09) were upregulated in the ASD individuals.

When the samples from the ASD individuals were examined for correlations between the expression of each miRNA and each of the three domains assessed by ADI-R (Domain A score, social; Domain score BV, communication; Domain C score, stereotype), none of the miRNA expressions was correlated with any of the domains (see Additional file 4).

The effects of age and sex on miRNA expression were examined by ANCOVA. The difference in the expression of miRNAs between the ASD and control groups remained significant even after adjusting for the effects of age and sex (see Additional file 5).

### Receiver operating characteristic analysis

**Figure 3:**
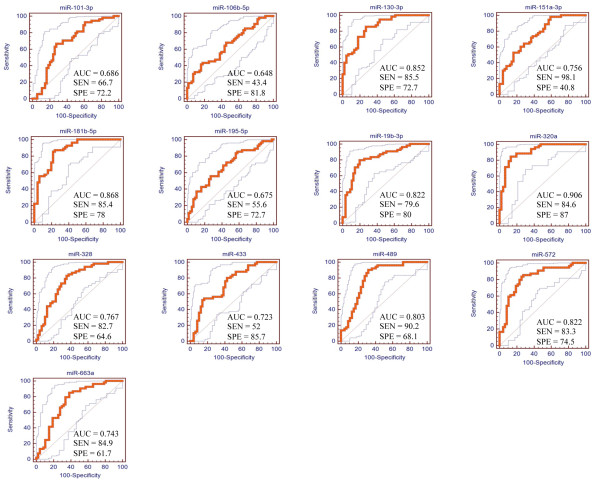
**Receiver operating characteristic (ROC) curve of 13 microRNAs.** AUC, area under the ROC curve; SEN, sensitivity; SPE, specificity.

Receiver operating characteristic (ROC) curve analysis was used to evaluate the predictive power of differentially expressed miRNAs to distinguish between ASD individuals and controls. The analysis showed significant diagnostic values of these 13 differentially expressed miRNAs for ASD (Figure 3). High values for sensitivity, specificity and the area under the curve (AUC) were observed for five miRNAs: miR-181b-5p, miR-320a, miR-572, miR-130a-3p and miR-19b-3p (see Additional file 6).

### Enrichment pathway analysis and target gene prediction

**Figure 4:**
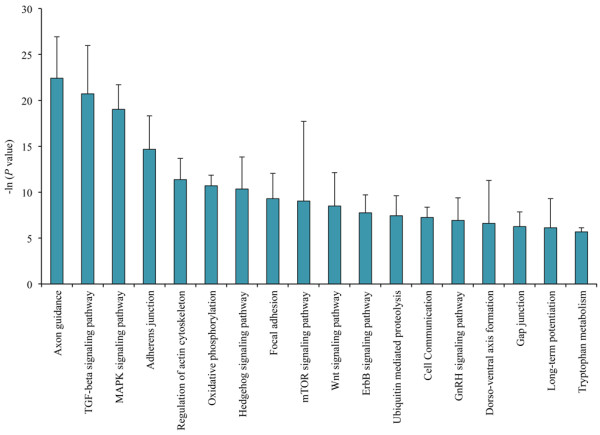
Neurologically relevant pathways (predicted by DIANA mirPath) of the target genes of differentially expressed microRNAs.

Using DIANA mirPath software, we found that the predicted target genes of the differentially expressed miRNAs could be involved in diverse vital neurological pathways (Figure 4). By a thorough analysis of the target genes and the pathways involving them, a total of 600 predicted genes and 18 neurological pathways were found (see Additional file 7). The top ten neurological pathways were those involved in axon guidance, TGF-beta signaling, MAPK signaling, adherens junction, regulation of actin cytoskeleton, oxidative phosphorylation, hedgehog signaling, focal adhesion, mTOR signaling and Wnt signaling. No specific pathways were observed for miR-572.

## Discussion

This is the first report on serum miRNAs in subjects with ASD. Altered expression of 13 miRNAs (downregulation of 8 miRNAs; upregulation of 5 miRNAs) was observed in our ASD subjects. Previous reports have shown a differential expression pattern of miRNAs in the postmortem brain [13] and in the lymphoblastoid cell lines of ASD individuals [14-16]. The results of the present and previous studies are summarized in Table 2, in which hsa-miR-181b-5p, hsa-miR-195-5p, hsa-miR-320a and hsa-miR-328 showed the same direction of regulation as in the brain [13] and lymphoblasts [14-16], while hsa-miR-106b-5p, hsa-miR-19b-30 and hsa-miR-663a did not. The reason for the latter differences in miRNA expression between the present and previous results is not known. The fact that the direction of alteration in the expression of hsa-miR-106b-5p in this study was the opposite of that reported in the previous postmortem study [13] suggests that the serum level of certain miRNAs may not reflect that in the brain, and thus that our findings should be treated with caution. However, it was interesting that hsa-miR-181b-5p and hsa-miR-328 in serum showed the same direction of regulation as in the brain. As mentioned above, serum miRNA expression is very stable, reproducible and resistant to RNase action [21]. In addition, an ANCOVA showed that confounding factors such as age and sex did not influence the results observed here. Therefore, hsa-miR-181b-5p and hsa-miR-328 in serum may become peripheral biomarkers reflecting the miRNA expression profile of individuals with ASD.

**Table 2:** Comparison of the results of the present study and previous autism microRNA studies

**miR ID**	**Present result**	**Previous report**	**Type of sample**	**Reference**
hsa-miR-106b-5p	↑	↓	Brain	[13]
hsa-miR-181b-5p	↓	↓	Brain, lymphoblastoid cell line	[13,16]
hsa-miR-195-5p	↑	↑	Lymphoblastoid cell line	[15]
hsa-miR-19b-3p	↑	↓	Lymphoblastoid cell line	[14]
hsa-miR-320a	↓	↓	Lymphoblastoid cell line	[14]
hsa-miR-328	↓	↓	Brain	[13]
hsa-miR-663a	↓	↑	Lymphoblastoid cell line	[14]

(↑), upregulated, (↓), downregulated.

ROC curve analyses showed significant diagnostic values of 13 differentially expressed miRNAs for ASD (Figure 3). High values for sensitivity, specificity and area under the curve (AUC) were observed for five miRNAs: miR-181b-5p, miR-320a, miR-572, miR-130a-3p and miR-19b-3p (see Additional file 6). Therefore, these five miRNAs may be potential candidates for circulating miRNA-based prediction of ASD.

MiRNA can influence gene silencing via translational repression or mRNA degradation [24]. This mRNA destabilization may alter several downstream pathways and induce several noticeable effects [25]. A number of neurologically relevant pathways and target genes were identified by our enrichment analysis, with most of the target genes being involved in multiple pathways. Collectively, these results predicted several neurologically relevant canonical pathways for the target genes of the five miRNAs (miR-130a-3p, miR-19b-3p, miR-320a, miR181b-5p, and miR-572) that showed a good discriminative power in ROC analysis. Most of these genes and pathways have already been implicated in the pathogenesis of ASD [4,26-30].

The differentially expressed miRNAs in this study, which included miR-101, miR-106b, miR-130a, miR-151a, miR181b, miR-328, miR-433, miR-489 and miR-572, were previously reported to have altered expression in schizophrenia [31-35], supporting the contention that ASD and schizophrenia share common neurobiological features [36].

The detection of clinically useful noninvasive biomarkers that could allow early intervention for ASD is an important goal in ASD research. As an initial step toward this goal, our results suggest that serum miRNAs could be potential peripheral biomarkers of ASD. A limitation of this study is that the samples came from ASD individuals ranging from 6 to 16 years old, although ASD is an early-onset disorder. Therefore, to accurately evaluate the diagnostic power of circulating miRNAs in ASD, further studies on subjects of lower age will be necessary. Another limitation of the study is that we used the same sample set in both the screening and validation. It would have been more informative if we had screened an independent sample set.

## Conclusions

This preliminary noninvasive study found a set of significantly differentially expressed miRNAs in the sera of children with ASD. The predicted target genes of these miRNAs were found to be associated with neurologically relevant pathways and functions.

## Abbreviations

ADI-R: Autism Diagnostic Interview-Revised; ANCOVA: analysis of covariance; ASD: autism spectrum disorder; AUC: area under the curve; cDNA: complementary DNA; CI: confidence interval; Ct: threshold cycle; DSM-IV-TR: Diagnostic and Statistical Manual, Fourth Edition, Text Revision; MAPK: mitogen-activated protein kinase; miRNA: microRNA; miRTC: miRNA reverse transcription control; mRNA: messenger RNA; mTOR: mammalian target of rapamycin; PPC: positive PCR control; qPCR: quantitative polymerase chain reaction; ROC: receiver operating characteristics; SCID: Structured Clinical Interview for DSM-IV; SD: standard deviation; TGF: transforming growth factor; UTR: untranslated region.

## Competing interests

The authors declare that they have no competing interests.

## Authors’ contributions

MMV and AA carried out the molecular genetics studies and drafted the manuscript. IT and KI participated in the sequence alignment and helped to draft the manuscript. KY and TW analyzed data and performed statistical analysis. TT, MT, and TS evaluated and diagnosed participants, and are involved in revising the manuscript critically for clinical contents. KS and NM conceived of the study, participated in its design and coordination, and helped to draft the manuscript. All authors read and approved the final manuscript.

## Supplementary Material

Additional file 1 miScript miRNA PCR Array Human Neurological Development and Disease (MIHS-107Z) 96 × 4 format. Click here for file

Additional file 2 384-Well Custom miScript miRNA PCR Array (CMIHS02055E) Template - 48 × 8 format. Click here for file

Additional file 3 **miRNA mature sequences with miRBase accession ID.** Click here for file

Additional file 4 **Correlation between miRNA expression and Autism Diagnostic Interview-Revised (ADI-R) scores.** Click here for file

Additional file 5 ANCOVA analysis for checking the effect of age, sex, disease status and interaction between sex/status of differentially expressed miRNAs. Click here for file

Additional file 6 Receiver operating characteristics (ROC) curve data showing the sensitivity and specificity of the 13 differentially expressed miRNAs. Click here for file

Additional file 7 Predicted neurological pathways with the number of target genes and pathway ID. Click here for file
