# Supplementary material for: Serum microRNA profiles in children with autism
Source: Mol Autism. 2014 Jul 30;5:40. doi: 10.1186/2040-2392-5-40 (PMC4132421; doi:10.1186/2040-2392-5-40)
Supplement: Additional file 3 — miRNA mature sequences with miRBase accession ID. [file 2040-2392-5-40-S3.docx]

**Additional file 3**: miRNA mature sequences with miRBase accession ID

| **miR Name** | **Accession ID** | **Mature Sequence** |
| --- | --- | --- |
| hsa-miR-101-3p | MIMAT0000099 | UACAGUACUGUGAUAACUGAA |
| hsa-miR-106b-5p | MIMAT0000680 | UAAAGUGCUGACAGUGCAGAU |
| hsa-miR-130a-3p | MIMAT0000425 | CAGUGCAAUGUUAAAAGGGCAU |
| hsa-miR-151a-3p | MIMAT0000757 | CUAGACUGAAGCUCCUUGAGG |
| hsa-miR-181b-5p | MIMAT0000257 | AACAUUCAUUGCUGUCGGUGGGU |
| hsa-miR-195-5p | MIMAT0000461 | UAGCAGCACAGAAAUAUUGGC |
| hsa-miR-19b-3p | MIMAT0000074 | UGUGCAAAUCCAUGCAAAACUGA |
| hsa-miR-27a-3p | MIMAT0000084 | UUCACAGUGGCUAAGUUCCGC |
| hsa-miR-320a | MIMAT0000510 | AAAAGCUGGGUUGAGAGGGCGA |
| hsa-miR-328 | MIMAT0000752 | CUGGCCCUCUCUGCCCUUCCGU |
| hsa-miR-433 | MIMAT0001627 | AUCAUGAUGGGCUCCUCGGUGU |
| hsa-miR-489 | MIMAT0002805 | GUGACAUCACAUAUACGGCAGC |
| hsa-miR-572 | MIMAT0003237 | GUCCGCUCGGCGGUGGCCCA |
| hsa-miR-663a | MIMAT0003326 | AGGCGGGGCGCCGCGGGACCGC |
| hsa-miR-103a-3p٭ | MIMAT0000101 | AGCAGCAUUGUACAGGGCUAUGA |
| hsa-miR-191-5p٭ | MIMAT0000440 | CAACGGAAUCCCAAAAGCAGCUG |
| hsa-let 7a-5p٭ | MIMAT0000062 | UGAGGUAGUAGGUUGUAUAGUU |
| hsa-miR-16-5p٭ | MIMAT0000069 | UAGCAGCACGUAAAUAUUGGCG |
| Syn-cel-39٭† |  | UCACCGGGUGUAAAUCAGCUUG |

٭Normalization controls, † *Caenorhabditis elegans* miR-39
