# Supplementary material for: Serum microRNA profiles in children with autism
Source: Mol Autism. 2014 Jul 30;5:40. doi: 10.1186/2040-2392-5-40 (PMC4132421; doi:10.1186/2040-2392-5-40)
Supplement: Additional file 4 — Correlation between miRNA expression and Autism Diagnostic Interview-Revised (ADI-R) scores. [file 2040-2392-5-40-S4.docx]

| **miR ID** | **ADIRA** | **ADIRBV** | **ADIRC** |
| --- | --- | --- | --- |
| **hsa-miR-101-3p** | 0.141 | 0.623 | 0.828 |
| **hsa-miR-106b-5p** | 0.275 | 0.47 | 0.899 |
| **hsa-miR-130a-3p** | 0.497 | 0.926 | 0.753 |
| **hsa-miR-151a-3p** | 0.602 | 0.719 | 0.215 |
| **hsa-miR-181b-5p** | 0.51 | 0.443 | 0.367 |
| **hsa-miR-195-5p** | 0.612 | 0.751 | 0.994 |
| **hsa-miR-19b-3p** | 0.668 | 0.49 | 0.945 |
| **hsa-miR-320a** | 0.533 | 0.63 | 0.368 |
| **hsa-miR-328** | 0.523 | 0.483 | 0.873 |
| **hsa-miR-433** | 0.106 | 0.531 | 0.85 |
| **hsa-miR-489** | 0.654 | 0.524 | 0.39 |
| **hsa-miR-572** | 0.148 | 0.301 | 0.119 |
| **hsa-miR-663a** | 0.183 | 0.221 | 0.656 |

**Additional File 7**: Correlation between miRNA expression and ADI-R scores.
