# Supplementary material for: Serum microRNA profiles in children with autism
Source: Mol Autism. 2014 Jul 30;5:40. doi: 10.1186/2040-2392-5-40 (PMC4132421; doi:10.1186/2040-2392-5-40)
Supplement: Additional file 5 — ANCOVA analysis for checking the effect of age, sex, disease status and interaction between sex/status of differentially expressed miRNAs. [file 2040-2392-5-40-S5.docx]

**Additional file 4:** ANCOVA analysis for checking the effect of age, gender, diseased status and interaction between gender /status of differentially expressed miRNAs.

| miR ID | Age - f (df) p | Gender - f (df) p | Status - f (df) p | Interaction (Gender / Status) - f (df) p | Final (corrected) p value |
| --- | --- | --- | --- | --- | --- |
| hsa-miR-101-3p | 2.825 (1,103) | 3.404 (1,103) | 3.755 (1,103) | 2.065 (1,103) | 0.055 |
| hsa-miR-106b-5p | 0.067 (1,103) | 3.642 (1,103) | 5.472 (1,103)⃰ | 0.085 (1,103) | 0.021 |
| hsa-miR-130a-3p | 0.371 (1,94) | 0.037 (1.94) | 29.485 (1,94)⃰ ⃰ ⃰ | 0.117 (1,94) | <0.001 |
| hsa-miR-151a-3p | 11.513 (1,97)⃰ ⃰ | 0.618 (1,97) | 16.520 (1,97)⃰ ⃰ ⃰ | 0.069 (1,97) | <0.001 |
| hsa-miR-181b-5p | 0.013 (1,99) | 0.456 (1,99) | 40.749 (1,99)⃰ ⃰ ⃰ | 0.425 (1,99) | <0.001 |
| hsa-miR-195-5p | 13.177 (1,104)⃰ ⃰ ⃰ | 0.817 (1,104) | 7.452 (1,104)⃰ ⃰ | 0.817 (1,104) | 0.007 |
| hsa-miR-19b-3p | 3.426 (1,104) | 1.552 (1,104) | 23.651 (1,104)⃰ ⃰ ⃰ | 0.061 (1,104) | <0.001 |
| hsa-miR-320a | 2.591 (1,93) | 4.017 (1,93) | 44.625 (1,93)⃰ ⃰ ⃰ | 0.727 (1,93) | <0.001 |
| hsa-miR-328 | 13.104 (1,95)⃰ ⃰ ⃰ | 3.681 (1,95) | 17.549 (1,95)⃰ ⃰ ⃰ | 0.630 (1,95) | <0.001 |
| hsa-miR-433 | 2.516 (1,94) | 0.980 (1,94) | 7.501 (1,94)⃰ ⃰ | 0.586 (1,94) | 0.007 |
| hsa-miR-489 | 7.594 (1,93)⃰ ⃰ | 0.537 (1,93) | 17.516 (1,93)⃰ ⃰ ⃰ | 0.005 (1,93) | <0.001 |
| hsa-miR-572 | 4.588 (1,96)⃰ | 7.441 (1,96)⃰ ⃰ | 20.787 (1,96)⃰ ⃰ ⃰ | 0.040 (1,96) | <0.001 |
| hsa-miR-663a | 13.144 (1,95)⃰ ⃰ ⃰ | 2.692 (1,95) | 10.984 (1,95)⃰ ⃰ | 0.486 (1,95) | 0.001 |

*⃰ p value <0.05, ⃰ ⃰ p value <0.01, ⃰ ⃰ ⃰ p value <0.001*
