# Supplementary material for: Serum microRNA profiles in children with autism
Source: Mol Autism. 2014 Jul 30;5:40. doi: 10.1186/2040-2392-5-40 (PMC4132421; doi:10.1186/2040-2392-5-40)
Supplement: Additional file 6 — Receiver operating characteristics (ROC) curve data showing the sensitivity and specificity of the 13 differentially expressed miRNAs. [file 2040-2392-5-40-S6.docx]

**Additional file 5:** ROC curve data showing the sensitivity and specificity of 13 differentially expressed miRNAs.

| **miR ID** | **AUC** | **Sensitivity (%)** | **Specificity (%)** | **95% CI** |
| --- | --- | --- | --- | --- |
| hsa-miR-101-3p | 0.686 | 66.7 | 72.2 | 0.5825 to 0.7886 |
| hsa-miR-106b-5p | 0.648 | 43.4 | 81.8 | 0.5446 to 0.7508 |
| hsa-miR-130a-3p | 0.852 | 85.5 | 72.7 | 0.7790 to 0.9259 |
| hsa-miR-151a-3p | 0.756 | 98.1 | 40.8 | 0.6640 to 0.8485 |
| hsa-miR-181b-5p | 0.868 | 85.4 | 78 | 0.7999 to 0.9360 |
| hsa-miR-195-5p | 0.675 | 55.6 | 72.7 | 0.5745 to 0.7757 |
| hsa-miR-19b-3p | 0.822 | 79.6 | 80 | 0.7419 to 0.9019 |
| hsa-miR-320a | 0.906 | 84.6 | 87 | 0.8456 to 0.9654 |
| hsa-miR-328 | 0.767 | 82.7 | 64.6 | 0.6718 to 0.8619 |
| hsa-miR-433 | 0.723 | 52 | 85.7 | 0.6221 to 0.8236 |
| hsa-miR-489 | 0.803 | 90.2 | 68.1 | 0.7118 to 0.8944 |
| hsa-miR-572 | 0.822 | 83.3 | 74.5 | 0.7377 to 0.9061 |
| hsa-miR-663a | 0.743 | 84.9 | 61.7 | 0.6434 to 0.8435 |

AUC = Area under the ROC Curve, CI = Confidence Interval
