# Supplementary material for: Serum microRNA profiles in children with autism
Source: Mol Autism. 2014 Jul 30;5:40. doi: 10.1186/2040-2392-5-40 (PMC4132421; doi:10.1186/2040-2392-5-40)
Supplement: Additional file 7 — Predicted neurological pathways with the number of target genes and pathway ID. [file 2040-2392-5-40-S7.docx]

| **Pathway Name** | **Pathway ID** | **No. of genes** | **-ln(p-value)** |
| --- | --- | --- | --- |
| Axon guidance | hsa04360 | 53 | 22.41 |
| TGF-beta signaling pathway | hsa04350 | 41 | 20.71 |
| MAPK signaling pathway | hsa04010 | 83 | 19.01 |
| Adherens junction | hsa04520 | 31 | 14.68 |
| Regulation of actin cytoskeleton | hsa04810 | 63 | 11.37 |
| Oxidative phosphorylation | hsa00190 | 4 | 10.69 |
| Hedgehog signaling pathway | hsa04340 | 23 | 10.36 |
| Focal adhesion | hsa04510 | 57 | 9.31 |
| mTOR signaling pathway | hsa04150 | 20 | 9.03 |
| Wnt signaling pathway | hsa04310 | 45 | 8.48 |
| ErbB signaling pathway | hsa04012 | 32 | 7.77 |
| Ubiquitin mediated proteolysis | hsa04120 | 40 | 7.45 |
| Cell Communication | hsa01430 | 10 | 7.25 |
| GnRH signaling pathway | hsa04912 | 31 | 6.94 |
| Dorso-ventral axis formation | hsa04320 | 13 | 6.62 |
| Gap junction | hsa04540 | 30 | 6.25 |
| Long-term potentiation | hsa04720 | 22 | 6.11 |
| Tryptophan metabolism | hsa00380 | 2 | 5.68 |

**Additional file 6**: Predicted neurological pathways with number of target genes and pathway ID.

-ln(p-value) = natural logarithm.
